# Benchmark evaluation in task and motion planning using iteratively deepened AND/OR graph networks

**DOI:** 10.3389/frobt.2026.1853007

**Published:** 2026-07-15

**Authors:** Hossein Karami, Antony Thomas, Fulvio Mastrogiovanni

**Affiliations:** 1 Department of Informatics, Bioengineering, Robotics, and Systems Engineering, University of Genoa, Genoa, Italy; 2 Robotics Research Center, IIIT Hyderabad, Hyderabad, India

**Keywords:** AND/OR graph networks, benchmark evaluation, iterative AND/OR graphs, task and motion planning, task planning

## Abstract

In robotics research, each subdomain presents a distinct set of challenges, and any framework designed for a given domain must effectively address these complexities. However, a single application within that domain may not fully capture the breadth of challenges inherent to it. To enable systematic and comprehensive evaluation, the robotics community has developed standardized problem scenarios and associated performance metrics, commonly referred to as benchmarks, which collectively represent the diverse challenges arising across applications. In this work, we evaluate our task–motion Planning (TMP) framework on five benchmarks proposed by the TMP community. We begin by briefly describing our iterative deepening AND/OR graph–based TMP planner. Subsequently, we assess its performance across these benchmarks, each designed to capture different aspects of the challenges in TMP. The evaluation demonstrates that the proposed planner successfully solves all five benchmarks, thereby indicating that our framework constitutes a robust and effective solution for TMP.

## 1 Introduction

Autonomous robots operating in the real world often need to sequence different actions to achieve their goals. When a single action cannot accomplish a goal, task planning algorithms facilitate searching for and ordering different actions to achieve the goal. However, each of these actions may need a feasibility check to ascertain its execution. For example, grasping a target of interest requires finding a feasible trajectory for the gripper. Such feasibility checks determine the geometric viability of the actions. If no viable path exists, then objects that hinder feasibility may need to be rearranged. Therefore, reasoning is required in both the discrete task domain and the continuous geometric or motion planning domain to decide the actions and their order of execution.

Task–motion Planning (TMP), that combines task planning and motion planning is an area of active research with applications, including manipulation (Kaelbling and Lozano-Pérez, 2013; Srivastava et al., 2014; Dantam et al., 2016), object rearrangement (Krontiris et al., 2014; Krontiris and Bekris, 2015; Karami et al., 2022), robot navigation (Lo et al., 2018; Thomas et al., 2021), and mobile manipulators (Garrett et al., 2018a; Garrett et al., 2018b). Each application exhibits different challenges of TMP, and a holistic TMP planner should be easily adaptable to cater to various applications.

The main contribution of this paper is the evaluation of different benchmarks proposed by the TMP community (Lagriffoul et al., 2018), which cover various challenging aspects of TMP. The evaluation is performed across five different benchmarks employing our TMP planner. As described in Section 4, our planner encodes the task-level abstractions of TMP efficiently and compactly within an AND/OR graph that grows iteratively until a solution is found. For motion planning, we use an off-the-shelf motion planner, and the benchmarks are evaluated in simulation using a PR2 robot. The evaluations presented in Section 5 show that our planner effectively implements all five benchmarks, thereby demonstrating that our framework constitutes a robust and effective solution for TMP.

## 2 Related work

In recent years, a wide range of TMP approaches have been developed for different application scenarios. The aSyMov planner (Cambon et al., 2009) has been demonstrated on mobile manipulation tasks, including a *forklift and boxes* scenario in which forklifts transport boxes to designated locations, as well as the classical Towers of Hanoi problem involving object rearrangement. Dornhege et al. (2009a) and Dornhege et al. (2009b) focus on mobile manipulation with experiments centered on block rearrangement tasks. The evaluation of parallel manipulators for assembly tasks is presented in Erdem et al. (2011). Additionally, a TMP framework that incorporates estimation and perception uncertainties is proposed in Kaelbling and Lozano-Pérez (2013), with a focus on mobile manipulators performing household tasks. Manipulation in clutter or rearrangement planning is the focus of the works in Dogar and Srinivasa (2020) and Srivastava et al. (2014). The planner in Lagriffoul et al. (2014) is shown to successfully execute different instances of *pick and place*, *filling a glass*, and *stacking cups*. Maximizing the height of a physically stable construction from a collection of different objects is demonstrated in Toussaint (2015). The IDTMP approach (Dantam et al., 2016) is validated on the Blocks World problem (rearranging three blocks from one configuration to another), along with multiple instances of rearrangement planning. The FFRob planner (Garrett et al., 2018a) is evaluated on different rearrangement planning scenarios. The planner also evaluates experiments in a kitchen scenario where a robot is required to prepare a meal. Real-world kitchen manipulation tasks in the presence of environmental uncertainty are demonstrated in Garrett et al. (2020a). A TMP approach to human–robot interaction is developed in Khandelwal et al. (2017). Navigation Among Movable Obstacles is the focus of the works in Stilman and Kuffner (2005) and Van Den Berg et al. (2009). TMP in the context of robot navigation is explored in Lo et al. (2018). Both single and multi-robot TMP for robot navigation incorporating state uncertainties are presented in Thomas et al. (2021) and Thomas et al. (2020).

Existing TMP approaches are often developed under specific modeling assumptions and are typically evaluated on a limited set of problem settings, focusing on particular aspects such as rearrangement, non-monotonicity, or geometric feasibility. As a result, their adaptability across diverse TMP scenarios is not always evident.

In this work, rather than proposing a new planning algorithm, we focus on systematically evaluating our previously developed AND/OR graph–based TMP framework (Karami et al., 2025) across a diverse set of community-standard benchmarks. Our formulation encodes task-level abstractions compactly within an AND/OR graph, enabling flexibility in representing different problem structures without redesigning the planner. We assess its performance on five benchmarks (Section 5) that capture complementary challenges in TMP, providing a unified evaluation of its robustness and generality.

## 3 Background

Task planning or classical planning is the process of finding a discrete sequence of actions from the current state to a desired goal state (Ghallab et al., 2016).

Definition 1 *A task domain* Ω *can be represented as a state transition system and is a tuple* $\Omega =\left\langle S,A,\gamma ,s_{0},S_{g}\right\rangle$ *where*:

-
S is a finite set of states;
-
A is a finite set of actions;
-
γ: S×A→S such that $s^{\prime }=\gamma \left(s,a\right)$ ;
-
$s_{0}\in S$ is the start state;
-
$S_{g}\subseteq S$ is the set of goal states.

Definition 2 *The task plan for a task domain* Ω *is the sequence of actions* $a_{0},\ldots ,a_{m}$ *such that* $s_{i+1}=\gamma \left(s_{i},a_{i}\right)$ *, for* i=0,…,m *and* $s_{m+1}$ *satisfies* $S_{g}$ .

Motion planning finds a sequence of collision free configurations from a given start configuration to a desired goal configuration (Latombe, 1991).

Definition 3 *A motion planning domain is a tuple* $M=\left\langle C,f,q_{0},G\right\rangle$ *where*:

-
C is the configuration space;
-
f={0,1} , for collision (f=0) else (f=1) ;
-
$q_{0}\in C$ is the initial configuration;
-
G∈C is the set of goal configurations.

Definition 4 *A motion plan for* M *finds a collision free trajectory in* C *from* $q_{0}$ *to* $q_{n}\in G$ *such that* f=1 *for* $q_{0},\ldots ,q_{n}$ *. Alternatively, A motion plan for* M *is a function of the form* $\tau :\left[0,1\right]\to C_{\mathrm{free}}$ *such that* $\tau \left(0\right)=q_{0}$ *and* τ(1)∈G *, where* $C_{\mathrm{free}}\subset C$ *is the configurations where the robot does not collide with other objects or itself.*

TMP combines discrete task planning and continuous motion planning to facilitate efficient interaction between the two domains. Below, we define the TMP problem formally.

Definition 5 *A task–motion planning with task domain* Ω *and motion planning domain* M *is a tuple* $\Psi =\left\langle C,\Omega ,\phi ,\xi ,q_{0}\right\rangle$ *where:*

-
$\phi :S\to 2^{C}$ , maps states to the configuration space;
-
$\xi :A\to 2^{C}$ , maps actions to motion plans.

Definition 6 *The TMP problem for the TMP domain* Ψ *is to find a sequence of discrete actions* $a_{0},\ldots ,a_{n}$ *such that* $s_{i+1}=\gamma \left(s_{i},a_{i}\right)$ *,* $s_{n+1}\in S_{g}$ *and a corresponding sequence of motion plans* $\tau_{0},\ldots ,\tau_{n}$ *such that for* i=0,…,n *, it holds that (1)* $\tau_{i}\left(0\right)\in \phi \left(s_{i}\right)\;and\;\tau_{i}\left(1\right)\in \phi \left(s_{i+1}\right)$ *, (2)* $\tau_{i+1}\left(0\right)=\tau_{i}\left(1\right)$ *, and (3)* $\tau_{i}\in \xi \left(a_{i}\right)$ .

We now provide a brief overview of AND/OR graphs. An AND/OR graph is a graph that represents a problem-solving process (Chang and Slagle, 1971).

Definition 7 *An AND/OR graph* G *is a directed graph represented by the tuple* G=⟨N,H⟩ *where:*

-
N *is a set of nodes;*
-
H *is a set of hyper-arcs.*

For a given AND/OR graph G , $H=\left\{h_{1},\ldots ,h_{m}\right\}$ , where $h_{i}$ is a many-to-one mapping from a set of child nodes to a parent node. In that sense, a hyper-arc induces a logical *AND* relationship between the child nodes/states, i.e., all the child states should be satisfied simultaneously to achieve the parent state. Similarly, a single parent node can be the codomain for different hyper-arcs $h_{i}$ . These hyper-arcs are in a logical *OR* with the parent node. Nodes without any successors or children are called the *terminal* nodes. The terminal nodes are either success nodes or failure node.

We now mathematically define an AND/OR graph network; a detailed treatment of the same can be found in (Karami et al., 2025).

Definition 8 *For an AND/OR graph* G=⟨N,H⟩ *, an augmented AND/OR graph* $G^{a}$ *is a directed graph represented by the tuple* $G^{a}=\left\langle N^{a},H^{a}\right\rangle$ *where:*

-
$N^{a}=\left\{N,n^{v}\right\}$ *with* $n_{v}$ *being the virtual node;*
-
$H^{a}=\left\{H,H^{v}\right\}$ *with* $H^{v}={\left\{h_{i}^{v}\right\}}_{1\leq i\leq |H^{v}|}$ *.*

Definition 9 *An AND/OR graph network* Γ *is a directed graph* Γ=⟨G,T⟩ *where:*

-
$G=\left\{G_{1}^{a},\ldots ,G_{n^{\prime }}^{a}\right\}$ *is a set of augmented AND/OR graphs* $G_{i}^{a}$ *;*
-
$T=\left\{t_{1},\ldots ,t_{n^{\prime }-1}\right\}$ *is a set of transitions such that* $G_{i+1}^{a}=t_{i}\left(G_{i}^{a}\right),\;1\leq i\leq n^{\prime }-1$ *.*

where $n^{\prime }$ is the total number of graphs in the network. Alternatively, $n^{\prime }$ is also the depth of the network.

## 4 TMP-IDAN

In this section, we briefly describe our approach TMP-IDAN (Task–Motion Planning using Iterative Deepened AND/OR Graph Networks) that utilizes AND/OR graph networks to compactly encode the task-level abstractions to reduce the overall task planning complexity. A comprehensive treatment of TMP-IDAN can be found in (Karami et al., 2025; Karami et al., 2022).

**FIGURE 1:**
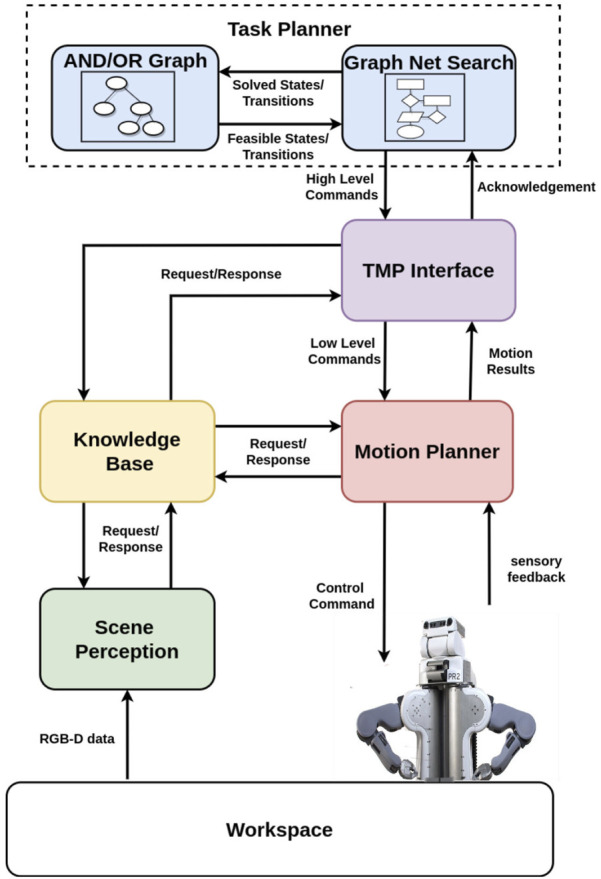
System’s architecture of the TMP-IDAN framework.

An exposition of TMP-IDAN is presented in Figure 1, illustrating its architectural components. The initial step involves the robot acquiring knowledge of its environment, which is achieved through the utilization of the *Scene Perception* module. This module is responsible for providing the *Knowledge Base* module with information about the current workspace configuration, that is, the location of the objects and the configuration of the robot. Evidently, the planning layer comprises two modules, namely, the *Task Planner* and *Motion Planner* modules. The *Task Planner* module embeds the augmented *AND/OR* graph and the AND/OR graph net search. The *Task Planner* provides a set of achieved transitions between the states to the graph net search module and receives the set of feasible states and transitions with the associated costs to follow. It then associates each state with an ordered set of actions to be executed. Once an action is carried out, it receives an acknowledgment and updates its internal structure to proceed with the next iteration of the network. The *TMP interface* serves as an intermediary connecting the task-level planner with the motion planning component. It takes high-level action directives from the *Task Planner* and translates them into the corresponding geometric parameters. For instance, a grasp action is converted into specific quantities such as the desired end-effector pose and the robot’s base configuration. These parameters are then forwarded to the *Motion Planner* to evaluate whether a valid motion exists. To perform this evaluation, the module accesses relevant details about the environment and robotic system from the *Knowledge Base*. If the action is deemed executable, it is issued for execution. After completion, the *Task Planner* is notified, and the *Knowledge Base* is updated to reflect the new state.

Algorithm 1 **TMP-IDAN.**

**Input:** $\left\langle C,\Omega ,\phi ,\xi ,q_{0}\right\rangle$ : Task-motion domain, $\left\langle N^{a},H^{a}\right\rangle$ : augmented AND/OR graph
1: **while** Goal not achieved **do**
2:  AddNewGraph()
3:  Request NextFeasibleStates()
4:  **if** Request = = empty **then**
5:   Go to Line 2
6:  **else**
7:   continue
8:  **end if**
9:  Find NextOptimalState()
10:  **for** (tasks, agents) in OptimalState **do**
   
▹ Agents can be robot arms or different robots
11:   SendToTMPI(tasks, agents)
12:   **for** task in tasks **do**
13:    **for** agent in agents **do**
14:     RequestKB()
      
▹ Call to *Knowledge Base*
15:     RequestSP()
      
▹ Call to *Scene Perception*
16:     RequestMP()
      
▹ Call to *Motion Planner*
17:    **end for**
18:   **end for**
19:  **end for**
20: **if** RequestMP() **then**
21:  Get OptimalMotionPlan()
22:  **if** OptimalMotionPlan executed **then**
23:   Go to Line 3
24:  **else**
25:   Go to Line 2
26:  **end if**
27: **else**
28:  Go to Line 9
29: **end if**
30: **end while**
31: **return** Task–Motion Plan

The overall planning procedure is outlined in Algorithm 1. The process starts from the initial configuration, where the *Task Planner* module initiates the creation of the AND/OR graph by invoking the *AddNewGraph* function (line 2). Subsequently, the planning phase proceeds by traversing the AND/OR graph (line 3) to identify the subsequent feasible state using the *NextOptimalState* subroutine. The tasks and agents (arms of the robot) of the feasible state are communicated to the *TMP interface* with the call to *SendToTMPI* (line 11). Upon completion of the aforementioned steps, the geometric coordinates of the objects, robot base, and grippers are transmitted to the TMP interface through the *Knowledge Base* and *Scene Perception* modules (lines 14–15). This exchange of information ensures that the TMP interface is supplied with the relevant and up-to-date data regarding the spatial arrangement of the components involved. The motion planner is then called to find a feasible trajectory to achieve the required state. If the motion plan execution fails, for example, due to a grasping failure, then a retry is attempted (line 25). If a motion plan is not found, the graph iterates to a new graph to try a new feasible state (line 9)—for example, moving an obstacle aside^1^. If the motion plan is successful and the goal is achieved, the algorithm terminates.

## 5 Benchmark evaluation

In this section, we evaluate the performance of TMP-IDAN on five benchmarks proposed in (Lagriffoul et al., 2014) as a method for assessing TMP planners. These benchmarks encompass a range of challenges commonly encountered in TMP problems. One such challenge arises when task actions become infeasible due to the absence of a viable motion plan. To address this, alternative task actions must be executed to resolve the geometric infeasibility. For instance, in scenarios involving cluttered environments, the target object may be surrounded by other objects that hinder the robot’s ability to grasp it. In such cases, it may be necessary to move some of these obstructing objects aside to create a clear path for picking up the target. With regard to the movement of obstructing objects, such scenarios may be categorized into two distinct problem classes. In monotonic problems, each object can be moved at most once, whereas in non-monotonic problems, each object can be moved multiple times. The presence of non-monotonicity introduces additional challenges, as previously achieved subgoals (such as moved objects) may need to be undone to fulfill subsequent objectives.

The categorization of different challenges associated with TMP problems is detailed in (Lagriffoul et al.), and each benchmark presented in the evaluation addresses a unique combination of these challenges. For the convenience of readers, we list these challenges below:

1. *Infeasible task actions*: Certain task actions may be rendered infeasible due to the absence of a corresponding motion plan. This infeasibility can stem from various factors, including the presence of blocking objects within the environment and the kinematic limits of the robot.
2. *Large task spaces*: This implies that the underlying task planning problem may require a significant amount of search effort.
3. *Motion/Task trade-off*: The problem can be solved with a reduced number of steps by carefully selecting appropriate grasps and object placements.
4. *Non-monotonicity*: Some objects may need to be moved more than once to achieve the goal.
5. *Non-geometric actions*: In this type of problem, a task action may change the discrete state Ω but not the configuration space.

### 5.1 Benchmark 1: Towers of Hanoi

This domain represents a modified version of the classical Towers of Hanoi problem. It involves moving disks, which are stacked in descending order of size, from one rod to another. The robot’s base remains fixed, and the three rods are positioned in a triangular formation. The disks are designed to be quite thick, and their arrangement may temporarily restrict the ability to pick up or place a disk on certain rods. This benchmark presents challenges such as a large task space and the occurrence of infeasible task actions.

For n disks, the optimal solution requires $2^{n}-1$ disk displacements. Thus, as the number of disks increases, the *task space* grows exponentially, yet, it is important to note that this solution does not consider geometric feasibility, and thus, there may be numerous *infeasible task actions*.

**FIGURE 2:**
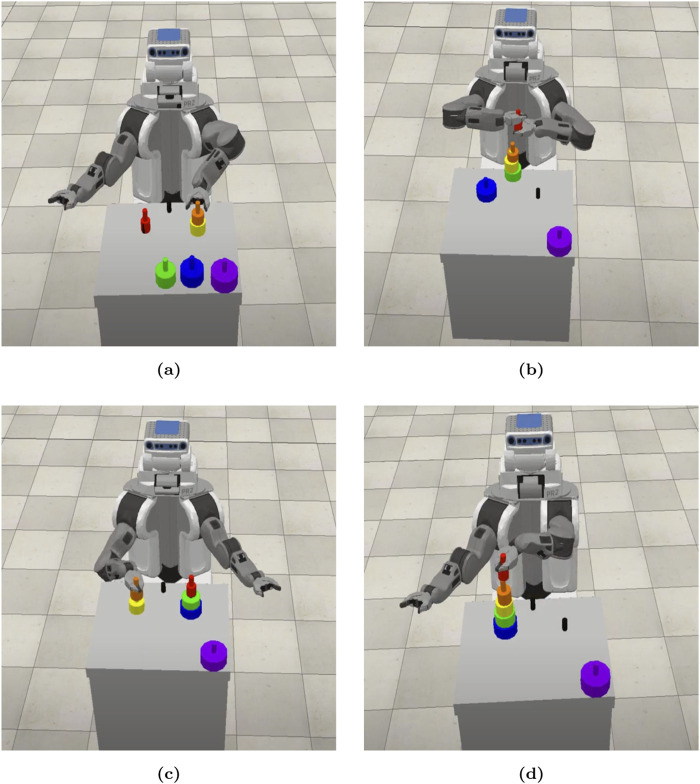
Illustration of a few steps involved in the Towers of Hanoi benchmark simulation: **(a)** The left manipulator grasps a disk and transfers it to a designated intermediate peg, **(b)** Due to the kinematic limitations of the left arm in reaching the destination peg, it hands over the disk to the right arm **(c)** The right arm positions the disk onto the left peg **(d)** All five disks are successfully placed onto the destination peg.

**FIGURE 3:**
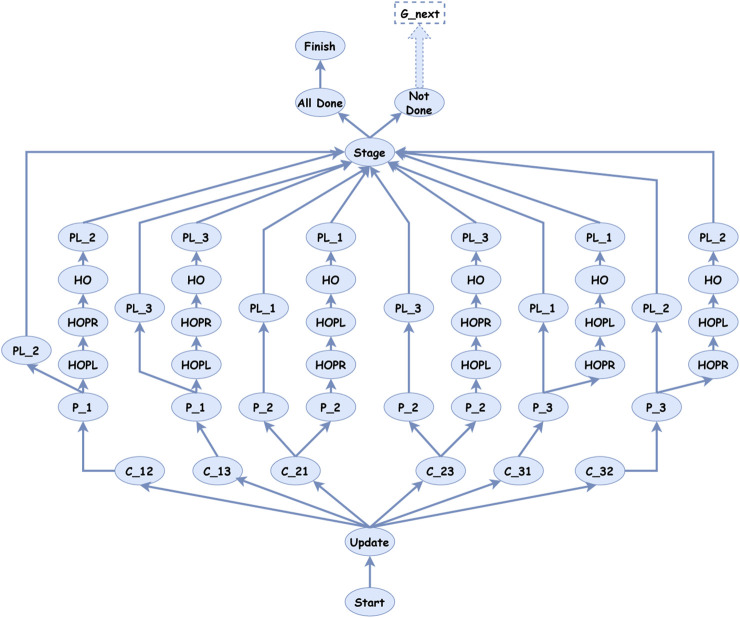
AND/OR graph for the Towers of Hanoi benchmark. C_ij : check pick from peg i and place onto peg j , P_i : pick disk from peg i , PL_i : place disk onto peg i , HOPL : hand-over and place disk on the left peg, HOPR : hand-over and place disk on the right peg, HO : hand-over.

To enhance the complexity of the problem, we conducted experiments involving a varying number of disks, ranging from three to six. A visualization of this benchmark is given in Figure 2. The right and left arms of the robot, with their corresponding grippers act as two different agents while the robot base is fixed. The AND/OR graph for this problem consists of 50 nodes and 53 hyper-arcs and is depicted in Figure 3. It should be noted that the depicted AND/OR graph is only one among the many possible graphs that can be utilized to solve this problem.

Statistics associated with different parameters and modules are given in Table 1. The second column in Table 1, that is, d, specifies the number of iterations of the AND/OR graph, representing the depth of the AND/OR graph network, required to reach the goal configuration. It should be noted that each AND/OR graph in the network essentially implements the movement of a single disk from one peg to another. Thus, for n disks, one would expect d to be $2^{n}-1$ . However, it is easily inferred that d is almost two times greater than the optimal solution of $2^{n}-1$ . As argued before, the observed increase in the number of iterations in the AND/OR graph is primarily attributed to the presence of geometrically infeasible actions. As discussed earlier, the rise in the number of iterations in the AND/OR graph is largely due to geometrically infeasible actions. In many cases, obstacles restrict feasible operations; for example, placing a disk on a rod can obstruct subsequent pick or place actions on other rods. Additionally, the kinematic constraints of the robot arms further limit direct transfers between rods. As a result, certain disk movements cannot be executed directly and necessitate the use of one or more intermediate pegs, thereby introducing additional AND/OR graph expansions.

**TABLE 1:** Statistics for the Towers of Hanoi benchmark. d -AND/OR graph network depth, TP-total task planning time, Right/Left MP-total motion planning time for the right/left arm, Right/Left MP attempts-number of motion planning attempts for the right/left arm.

Objects	d	TP [s]	Right MP [s]	Right MP attempts	Left MP [s]	Left MP attempts
3	16.67 ± 3.51	0.78 ± 0.01	105.67 ± 18.10	76.06 ± 0.74	111.37 ± 12.25	88.10 ± 4.43
4	31 ± 8.18	3.27 ± 0.37	245.64 ± 47.92	200.22 ± 20.09	186.65 ± 15.80	129.3 ± 0.29
5	61 ± 0.00	15.2 ± 0.61	458.24 ± 1.86	442.7 ± 20.31	409.75 ± 14.49	315.90 ± 16.31
6	140 ± 2.34	59.73 ± 3.50	1017.30 ± 55.43	854.15 ± 45.23	678.68 ± 35.87	475.5 ± 22.47

### 5.2 Benchmark 2: Blocks World

**FIGURE 4:**
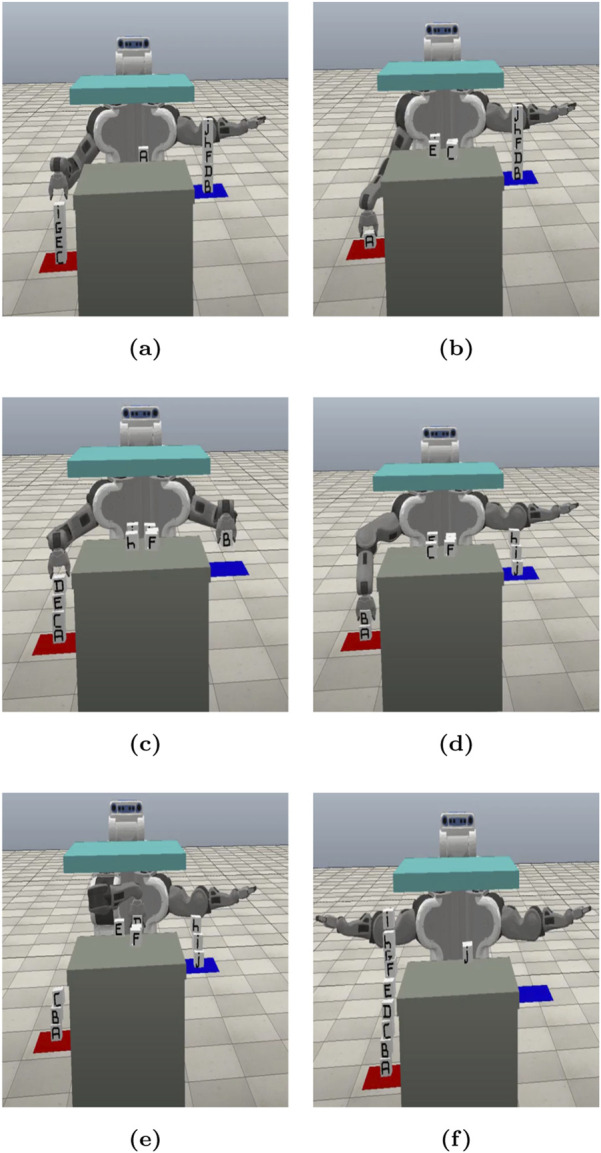
Illustration of various steps involved in the Blocks World benchmark: **(a)** Robot starts moving the blocks from the red tray onto the table, **(b)** Cubes from the red tray (except cube A) are placed on the table, **(c)** While the left arm moves the cubes from the blue tray onto the table to pick cube B, the right arm places cubes on the red tray to create enough space on the table, **(d)** Cube B is placed on the red tray **(e,f)** The process continues until the blocks are placed in order on the red tray.

The objective of this benchmark is to stack blocks/cubes in alphabetical order on the red tray as shown in Figure 4. To evaluate this benchmark, we employ 10 cubes labeled with letters from the English alphabet A through J . The robot base remains fixed throughout the task, and cube hand-over between the robot arms is not permitted.

Initially, both the red and blue trays contain stacks of cubes. Consequently, the manipulator needs to strategically place a subset of the blocks on the table and subsequently transfer them to the other tray. Notably, there is an obstacle in the form of a shelf above the table, prohibiting the stacking of one cube on top of another. Additionally, only top grasps are permitted for both cube picking and placement.

This benchmark exhibits different challenges, such as *task/motion planning trade-off*, *infeasible task actions,* and *large task spaces*. The task/motion planning trade-off involves striking a balance between fewer steps and a cluttered table, or many steps and an uncluttered table. Infeasible task actions may arise due to the limited grasping options or the presence of the shelf obstacle. Moreover, the different number of possible task configurations and sequences in this benchmark contributes to the large task space, posing additional complexities for the planner.

**FIGURE 5:**
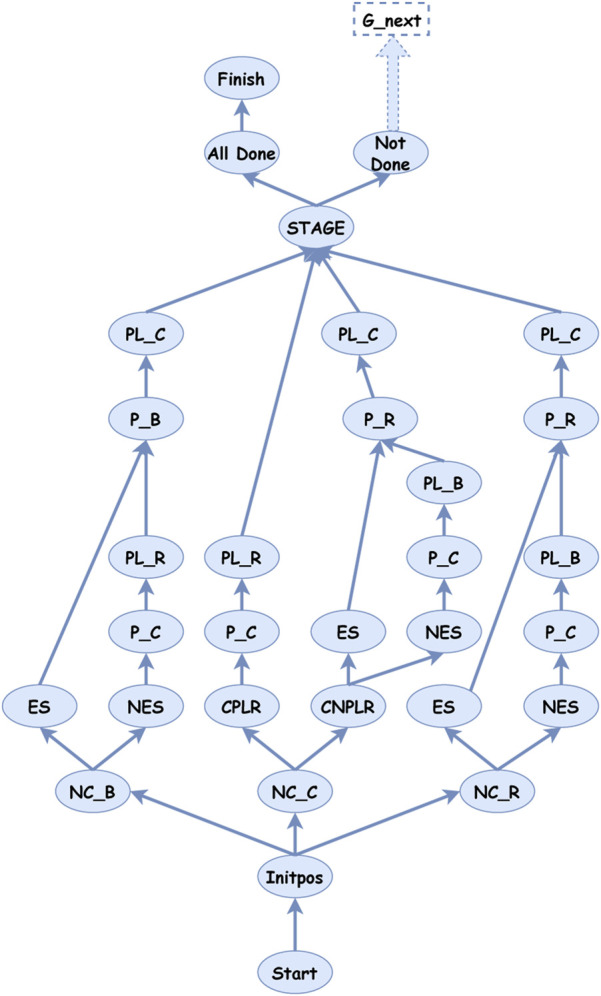
AND/OR graph developed for the Blocks World benchmark. location: { C : center, R : red tray, B : blue tray}, P_location : pick cube from location , PL_location : place cube onto location , ES : check if there is enough space, NES : not enough space, NC_location : next cube is in location , CPLR : cube can be placed on the red tray, CNPLR : cannot be placed on red tray.

Figure 5 shows the structure of the AND/OR graph we adopted to implement this benchmark. This AND/OR graph has 31 nodes and 35 hyper-arcs. Statistics of all the planner modules are listed in Table 2 and as can be seen from the table, on average 74 graphs are required to sort 10 cubes in alphabetical order. We note here that each AND/OR graph corresponds to the displacement of a single cube either from the tray to the table or *vice versa*. By examining the last four rows of the table, it is evident that the right arm is more extensively utilized than the left arm. This discrepancy is expected as the destination tray is positioned adjacent to the right arm, naturally requiring its increased involvement in the task execution. Motion planning failures due to actuation errors and grasping failures lead to replanning, adding to the depth of the network and thereby resulting in the high standard deviation value (see row 1, Table 2).

**TABLE 2:** Statistics of various modules of TMP-IDAN for the Blocks World benchmark, reported as the mean and standard deviation over 10 independent runs.

Module	Average	Standard deviation
Depth of AND/OR network	74	12.72
AND/OR graph	10.30 [s]	0.44 [s]
Graph search	1.16 [s]	0.004 [s]
Right arm plan	471.3 [s]	0.18 [s]
Left arm plan	257.37 [s]	10.23 [s]
Right arm plan attempt	327.91 [s]	11.23 [s]
Left arm plan attempt	176.26 [s]	8.13 [s]

### 5.3 Benchmark 3: sort clutter

The objective of this problem is to relocate all the N blue blocks to the left table and all the N green blocks to the right table, as depicted in Figure 7. Initially, both the left and right tables are empty, while the blocks are positioned on two separate tables. Additionally, there are 2N red blocks that serve as obstacles, hindering access to the blue and green blocks.

The challenge lies in efficiently navigating around the red blocks to reach and manipulate the desired blue and green blocks. This aspect of the problem highlights the infeasible task action criteria. The task involves planning and executing appropriate actions to overcome the obstacles, ensuring that the blue blocks are exclusively moved to the left table and the green blocks are exclusively moved to the right table. Therefore, the problem may lead to a large task space as the number N increases.

**FIGURE 6:**
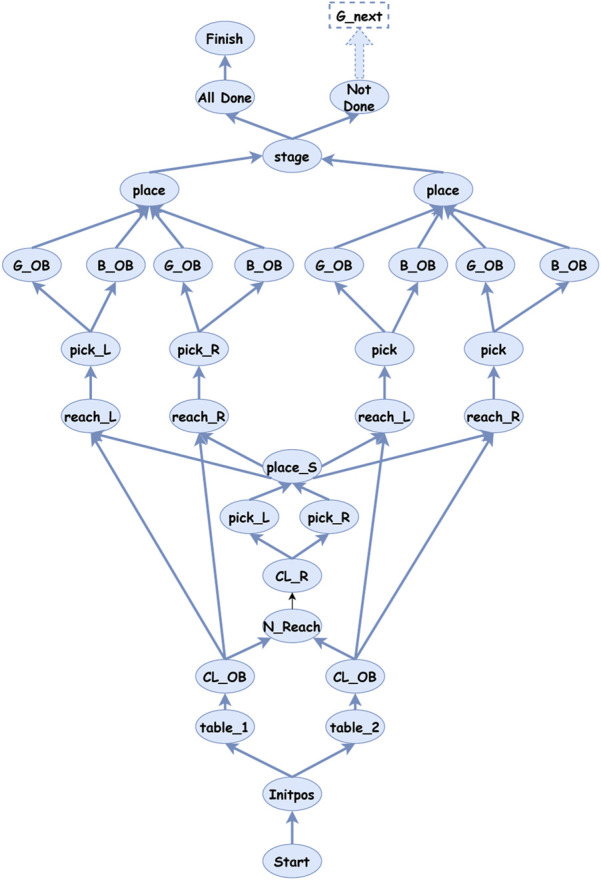
AND/OR graph employed for the implementation of the sort clutter benchmark. CL_OB : Closest object, this can be either a blue block or a green block, N_Reach : object is not reachable, reach_L : object is reachable by left arm, reach_R : object is reachable by the right arm, CL_R : Closest red object/block, G_OB object grasped is green, B_OB object grasped is blue, place : place grasped object onto its corresponding goal table, place_S : place the object on the same table.

The AND/OR graph used to implement this benchmark contains 33 nodes and 45 hyper-arcs. A visual representation of this graph is given in Figure 6. The provided graph corresponds to moving a blue or green block from a table and placing it on its respective goal table. If either a blue or a green block is occluded, the occluding red block is moved out of the way. The graph is iteratively expanded until all the blue blocks and the green blocks are placed on the left and right tables, respectively. Additionally, in this benchmark, the robot base is not static.

**FIGURE 7:**
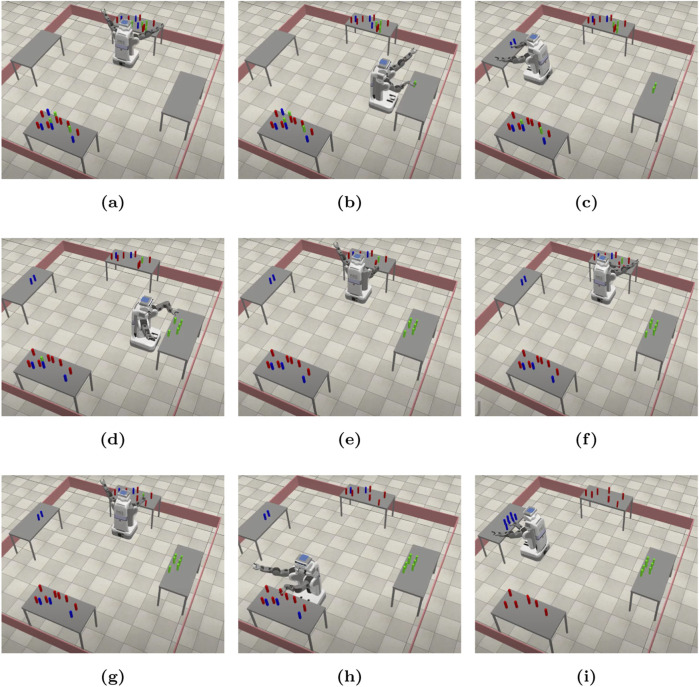
Illustration of different stages of the sort clutter benchmark. **(a)** start position of the robot. **(b–h)** Snapshots of different instances during the execution of TMP-IDAN framework, where blue blocks are being moved to the left table and the green blocks are being moved to the right table. **(i)** Task is completed.

The overall approach is summarized in Figure 7. As shown in Figure 7a, the robot following the AND/OR graph in Figure 6 moves to the table with a greater number of blue and green blocks (in case of equal numbers of blocks on both tables, the nearest table is selected). Once the robot nears a table, the closest block to the robot is selected for movement. If the block is red, it is moved aside to make way for grasping a blue or green block. If the selected block is either a blue block or a green block, the robot grasps the block and moves toward the corresponding goal table to place it. A case where a green block is placed on the right table is shown in Figure 7b. This procedure persists, and different stages of the experiment are shown in Figures 7c,d. Up until this point, the robot has been able to freely pick the green and blue blocks without any hindrance from the red blocks. In Figures 7e,f, the robot moves aside two red blocks that hinder the grasping of a green block. The corresponding green block is picked by the robot as seen in Figure 7g. In Figure 7h, the red blocks act as obstacles to the remaining blue blocks on the table. Finally, as shown in Figure 7i, all the blue and green blocks are sorted and placed on their respective tables.

In our experiments, we use N=7 and, as seen in Table 3, on average, 44.5 graphs are required to move the 7 blue and 7 green blocks to their respective tables. Note that, ideally, we should have 14 graphs since there are 14 blocks to be moved in total. As pointed out before, the red blocks act as obstacles, and moving a red block out of the way leads to a new graph, explaining the large number of graphs required. The planning time and attempt time for both the left and the right arm are almost the same, implying an equal contribution in sorting the blocks.

**TABLE 3:** Module statistics of TMP-IDAN for the sort clutter benchmark, reported as the mean and standard deviation over 10 independent runs.

Module	Average	Standard deviation
Depth of AND/OR network	44.5	3.53
AND/OR graph	16.46 [s]	2.61 [s]
Graph search	1.15 [s]	0.123 [s]
Right arm plan	275.07 [s]	27.54 [s]
Left arm plan	325.56 [s]	37.83 [s]
Base plan	93.8 [s]	4.65 [s]
Right arm plan attempt	205.11 [s]	16.89 [s]
Left arm plan attempt	279.45 [s]	100.37 [s]
Base plan attempt	90.18 [s]	3.07 [s]

### 5.4 Benchmark 4: non-monotonic

**FIGURE 8:**
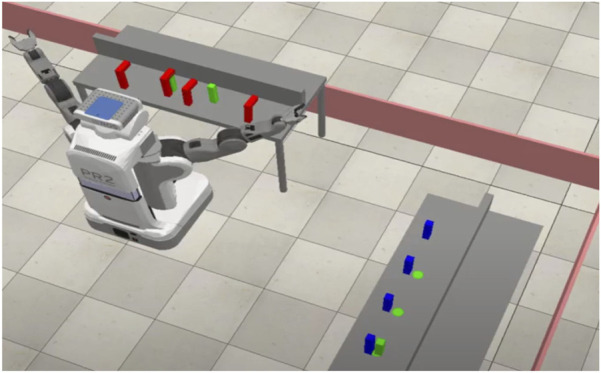
Non-monotonic benchmark environment.

The objective of this benchmark is to move all green blocks, as shown in Figure 8, from the left table to the right table, positioning them at their designated locations (indicated by green blobs in the figure) on the right table. Notably, the green blocks situated on the left table are obstructed by four red blocks, while the goal locations for these green blocks on the right table are impeded by four blue blocks. Thus, we have a case of *infeasible task actions*. In the goal state of this benchmark, the green blocks must be appropriately positioned on the right table, while simultaneously ensuring that the red and blue blocks are restored to their original locations. This task necessitates temporarily moving the obstructing red and blue blocks away from their initial positions to facilitate the picking up and placing of the green blocks. Subsequently, after the green blocks have been relocated, the red and blue blocks must be accurately returned to their original locations. This demonstrates the non-monotonic nature of the problem.

**FIGURE 9:**
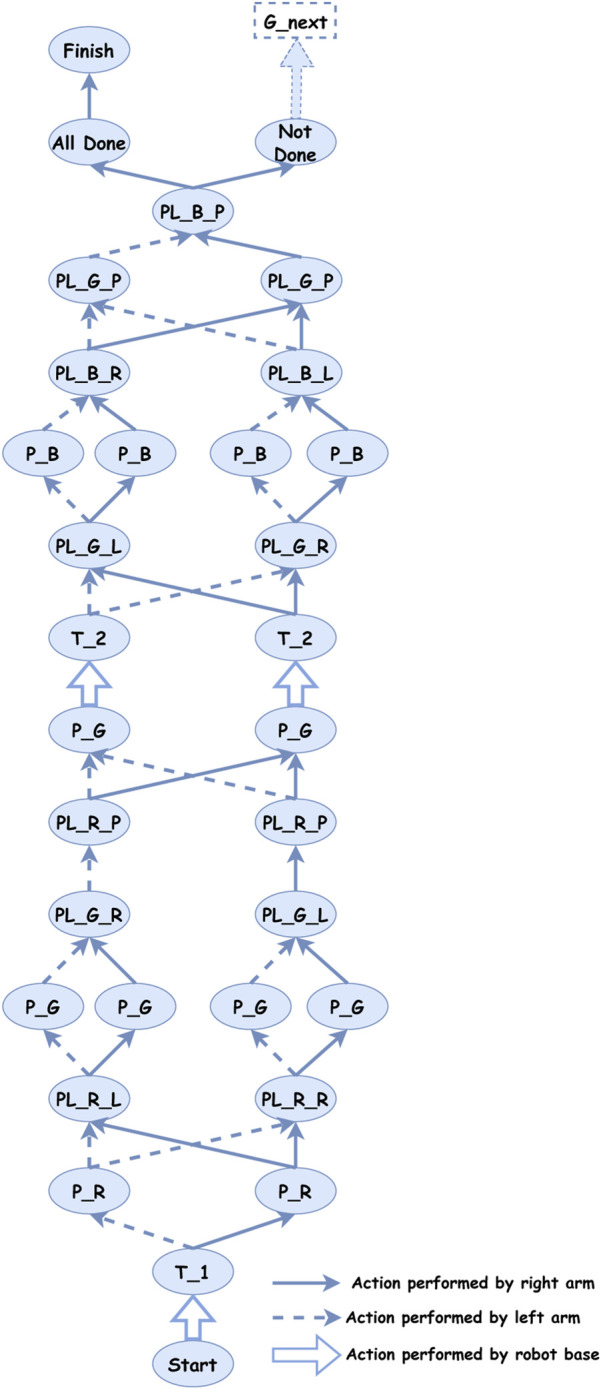
AND/OR graph synthesized for the non-monotonic benchmark. location: { R : right, L : left, P : destination}, color: { R : red, B : blue, G : green}, P_color : pick up the object of color color , PL_color_location : place the object of color color onto location , T_i : position of table i .

**FIGURE 10:**
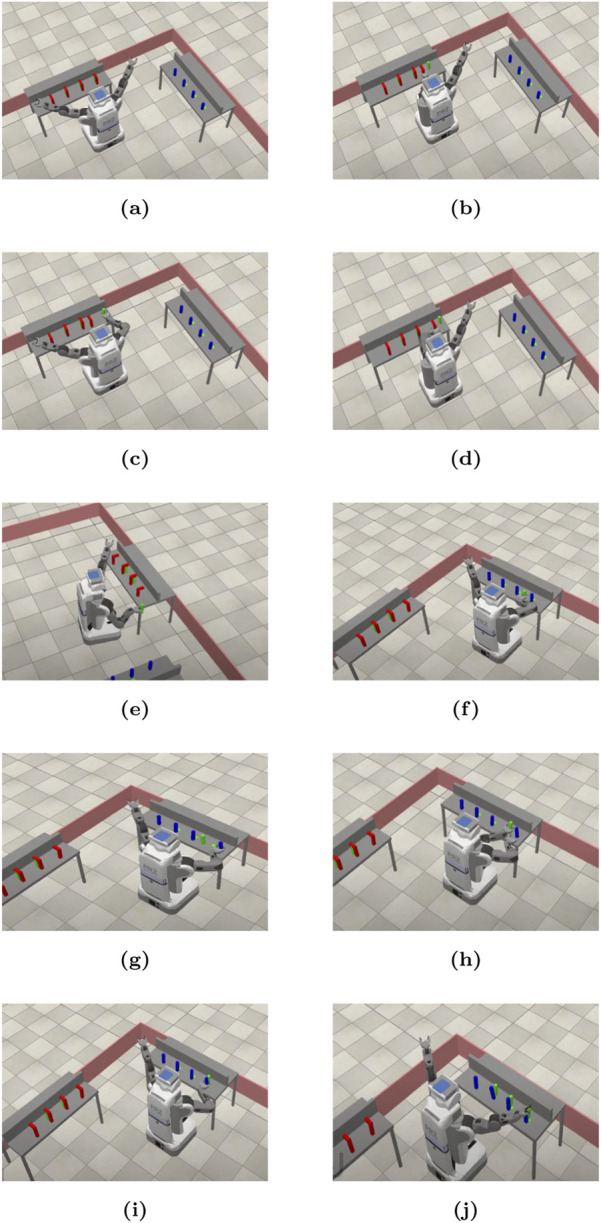
Illustration of various steps involved in the implementation of the non-monotonic benchmark. **(a)** start configuraiton, **(b–i)** different stages during execution, where the green blocks from the left table are moved to the right table, to their corresponding designated locations on the right table. **(j)** Goal state reached.

The AND/OR graph used to implement this benchmark (see Figure 9) has 34 nodes and 40 hyper-arcs. Figure 10 depicts the steps involved in implementing this benchmark. The start state of the problem as described above can be seen in Figure 10a. Subsequently, the robot proceeds toward the left table and moves a red block aside, which makes the green block graspable as shown in Figure 10b. The side of the table is determined based on the proximity of the robot’s arm to the blocks. Once a green block becomes reachable, it is moved aside so that the displaced red block may be placed back to its initial location. These stages are shown through Figures 10c,d. The green block is then picked up (see Figure 10e) and transported to the right table, and placed either to the left or right side (chosen randomly) of a blue block as shown in Figure 10f. The blue block is then moved aside (see Figure 10g), and the green block is placed in its goal position (Figure 10h). Afterward, the blue block is then moved back to its initial location, as can be seen in Figure 10i. The described procedure continues, and the final goal configuration of the benchmark is achieved, as can be seen in Figure 10j. Performance statistics of the different modules of TMP-IDAN are summarized in Table 4, averaged over 10 independent runs.

**TABLE 4:** Statistics of various modules involved in the implementation of the non-monotonic benchmark.

Module	Average	Standard deviation
Depth of AND/OR network	5.5	0.7
AND/OR graph	1.73 [s]	0.07 [s]
Graph search	0.38 [s]	0.18 [s]
Right arm plan	192.36 [s]	17.29 [s]
Left arm plan	276.71 [s]	59.38 [s]
Base plan	18.08 [s]	3.08 [s]
Right arm plan attempt	131.61 [s]	5.76 [s]
Left arm plan attempt	208.92 [s]	44.74 [s]
Base plan attempt	16.83 [s]	3.57 [s]

### 5.5 Benchmark 5: kitchen

In the kitchen domain, the main objective is meal preparation, which includes tasks such as cleaning glasses, cooking vegetables, and arranging the table. Glasses and vegetables are abstracted using blocks for representation. Specifically, the domain considers two types of vegetables: cabbages, represented as green blocks and intended for cooking, and radishes, represented by pink blocks.

The initial configuration, as shown in Figure 12a, depicts the radishes (pink blocks) obstructing the cabbages (green blocks), impeding the robot’s ability to pick them up. The problem thus exhibits the infeasible task action criteria. Thus, the radishes must be moved aside to cook the cabbages. As part of the benchmark requirements, to uphold a tidy kitchen environment, the robot must also restore the radishes to their original locations after the meal preparation is completed, introducing the non-monotonicity challenge. Cleaning the objects is accomplished by placing them in the dishwasher, while cooking is achieved by placing them in the microwave. Notably, the cook and clean actions are non-geometric in nature.

**FIGURE 11:**
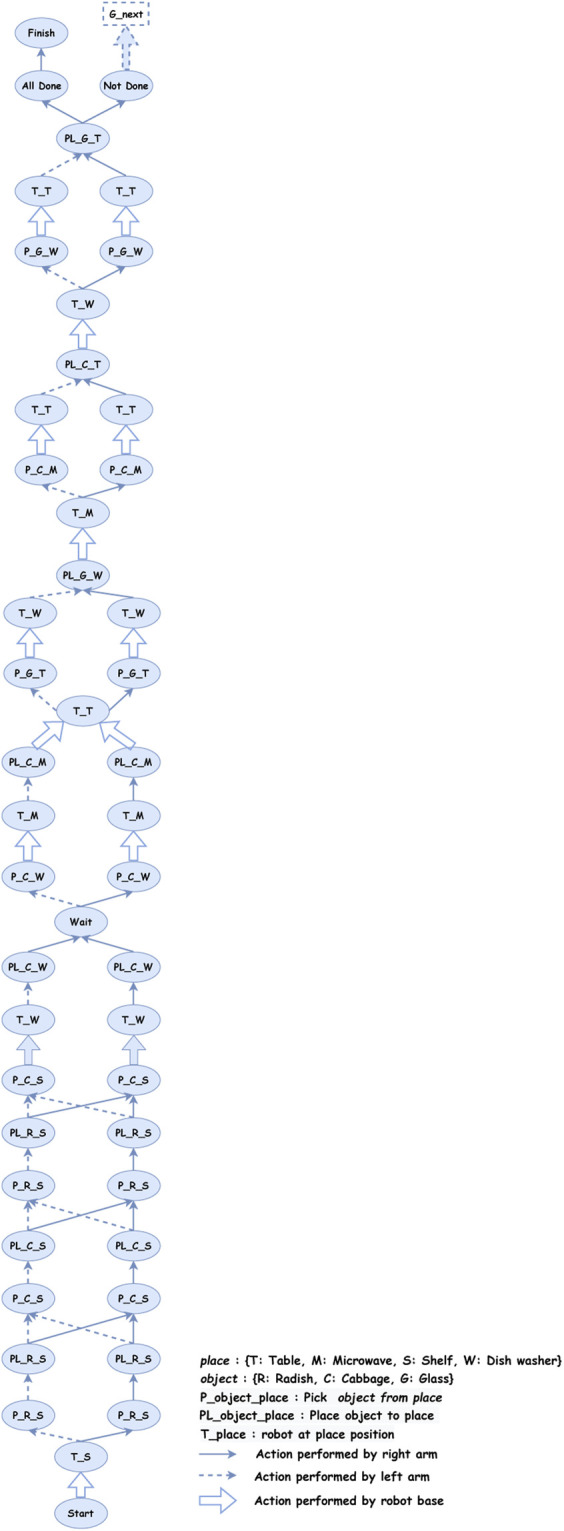
AND/OR graph used to implement the kitchen benchmark.

The AND/OR graph implementation for this benchmark involves 48 nodes and 60 hyper-arcs (see Figure 11). Table 5 summarizes the performance metrics of the different modules of TMP-IDAN across 10 independent runs. It is observed that the benchmark on the average iterates through 13.5 AND/OR graphs.

**TABLE 5:** Statistics of different modules of TMP-IDAN involved in the kitchen benchmark implementation.

Module	Average	Standard deviation
Depth of AND/OR network	13.5	4.95
AND/OR graph	7.78 [s]	0.47 [s]
Graph search	0.47 [s]	0.006 [s]
Right arm plan	317.89 [s]	9.52 [s]
Left arm plan	121.76 [s]	20.95 [s]
Base plan	56.86 [s]	0.017 [s]
Right arm plan attempt	284.06 [s]	29.07 [s]
Left arm plan attempt	84.11 [s]	12.76 [s]
Base plan attempt	57.83 [s]	1.62 [s]

**FIGURE 12:**
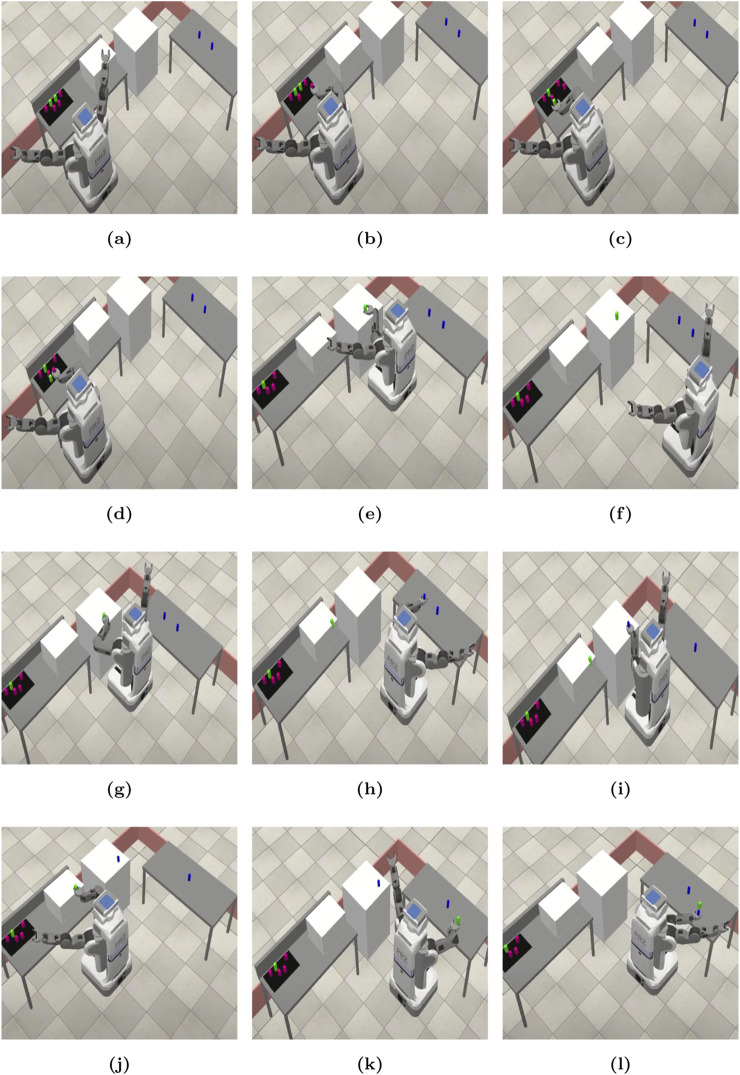
**(a)** The robot navigating to the vegetable shelf, **(b)** radish that obstructs access is removed. **(c)** The cabbage is picked to be tranferred. **(d,e)** radish is moved back to its inital locaiton and then the cabbage is placed in the dishwasher for cleaning. **(f)** Te robot simulates a waiting period by moving away and returning **(g–i)** the cleaned cabbage is moved to the microwave for cooking and then the robot picks up a glass and places it in the dishwasher for cleaning. **(j,k)** After the cabbage is cooked, it is taken from the microwave and placed on the dining table. **(l)** The cleaned glass is then taken from the dishwasher and positioned alongside the cabbage, completing the task.

Figure 12 illustrates the complete workflow for the kitchen benchmark. Initially, the robot navigates to the vegetable shelf (Figure 12a), where it removes a radish that obstructs access and temporarily relocates it to a free spot on the shelf (Figure 12b). It then grasps a cabbage and transfers it to a designated location (Figure 12c). To maintain organization, the previously moved radish is returned to its original position (Figure 12d). Before cooking, the cabbage is first placed in the dishwasher for cleaning (Figure 12e). During this stage, the robot simulates a waiting period by moving away and returning (Figure 12f). Subsequently, the cleaned cabbage is moved to the microwave for cooking (Figure 12g). While the cooking process is underway, the robot picks up a glass and places it in the dishwasher for cleaning (Figures 12h,i). After the cabbage is cooked, it is retrieved from the microwave (Figure 12j) and placed on the dining table (Figure 12k). The cleaned glass is then taken from the dishwasher and positioned alongside the cabbage (Figure 12l). This sequence is repeated until all required items, including the remaining cabbage and glass, are arranged on the table for serving.

## 6 Discussion

This section discusses the main implications of the benchmark evaluation, the relation between TMP-IDAN and representative TMP approaches, and the limitations that should be considered when interpreting the reported results.

A first observation concerns the role of the proposed evaluation. The aim of this paper is not to claim that TMP-IDAN is uniformly superior to existing TMP techniques, but rather to assess whether the same planning framework can be instantiated across a heterogeneous set of benchmark domains without changing the underlying planning principle. The five benchmarks considered in Section 5 cover complementary TMP challenges, including infeasible task actions, large discrete task spaces, task-motion trade-offs, non-monotonicity, and non-geometric actions. The fact that TMP-IDAN can solve all five benchmarks suggests that the AND/OR graph network representation provides a flexible abstraction layer for encoding different combinations of discrete and geometric constraints. In this sense, the main strength of TMP-IDAN is its adaptability across structurally different TMP problems, rather than its dominance in any single benchmark.

To better contextualize these results, we compare TMP-IDAN with representative TMP approaches such as IDTMP (Dantam et al., 2016), FFRob (Garrett et al., 2018a), and PDDLStream (Garrett et al., 2020b). A fully controlled quantitative comparison is difficult because these methods are usually evaluated on different domains, assumptions, platforms, and performance metrics. Moreover, the availability of identical implementations, identical low-level planners, and identical geometric models is rarely guaranteed across TMP systems. For this reason, the following comparison should be understood as a qualitative discussion of modeling assumptions and expected computational behavior, rather than as a direct ranking.

IDTMP addresses the tight integration of task and motion planning through an incremental constraint-based formulation. Its main advantage is the ability to backtrack when a discrete action is found to be geometrically infeasible, thereby supporting non-monotonic rearrangement problems. This is closely related to some of the challenges considered in our evaluation, especially those involving temporarily displaced objects that must later be restored. However, IDTMP has primarily been demonstrated on structured rearrangement-style domains. TMP-IDAN differs in that non-monotonicity and geometric infeasibility are encoded through the iterative expansion of an AND/OR graph network: when the current graph does not yield a feasible transition, a new graph is added, and alternative decompositions are explored. This makes the handling of infeasible task actions explicit at the level of the graph network evolution.

FFRob combines symbolic task planning with sampling-based motion planning by exploiting optimistic planning over sampled geometric instances. This formulation is particularly effective when a useful set of sampled placements, grasps, and motions can be generated and reused across the search. However, in domains with extensive stacking, repeated object relocation, or large combinatorial branching, the number of relevant sampled action instances may grow rapidly. This issue is particularly relevant for benchmarks such as Towers of Hanoi and Blocks World, where the symbolic search space expands quickly with the number of objects and where many nominally valid task actions may still be geometrically infeasible. In TMP-IDAN, the task-level structure is not generated by grounding all possible symbolic-geometric combinations in advance. Instead, the AND/OR graph compactly represents task alternatives, while motion planning is invoked only for candidate transitions selected during graph traversal. This allows the planner to focus geometric reasoning on the currently relevant portion of the search space, although repeated failures may still increase the depth of the AND/OR graph network.

PDDLStream provides a general and expressive integration of symbolic planning with black-box samplers through the notion of *streams*. Its strength lies in the ability to represent streams, that is, geometric, kinematic, and other continuous constraints within a symbolic planning framework while delaying sampling until needed. This makes PDDLStream highly flexible and applicable to many manipulation domains. At the same time, as the number of objects, streams, and possible action groundings increases, the number of stream evaluations may become a limiting factor. This is particularly relevant in cluttered domains, where many candidate object poses, grasps, and placements may need to be considered before a feasible plan is found. TMP-IDAN addresses the same general need for integrating symbolic and geometric reasoning, but does so through an explicitly structured AND/OR graph network rather than through a PDDL-level stream abstraction. The resulting representation is less domain-independent than PDDLStream in the sense that the AND/OR graph must be designed for the benchmark structure, but it offers a compact and interpretable encoding of the relevant task decomposition.

This point, however, highlights an important trade-off in our framework. TMP-IDAN does not eliminate the need for domain modeling. For each benchmark, an AND/OR graph must be specified so that the relevant task decompositions, alternatives, and recovery behaviors are represented. This design effort is visible in the benchmark-specific graphs reported in Section 5. However, once such a graph is specified, the same iterative deepening mechanism, graph traversal procedure, and task-motion interface can be used across domains. Thus, TMP-IDAN shifts part of the modeling burden from a fully grounded symbolic planning domain to a compact graph-based representation of task structure. This can be advantageous when the benchmark admits a natural hierarchical or recursive decomposition, but it may be less convenient in domains where the relevant task structure is not known in advance or changes substantially at run time.

The results also clarify where the computational cost of TMP-IDAN arises. Across the benchmarks, the time spent in graph construction and graph search is generally smaller than the time spent in motion planning. This is expected because the AND/OR graph operates at the discrete abstraction level, while the motion planner must reason over the continuous configuration space of the robot, including arm motions, base motions, grasp feasibility, and collision avoidance. The depth of the AND/OR graph network is therefore an important indicator of how often the planner must revise or extend its current task-level hypothesis due to geometric infeasibility, occlusion, or execution failure. For instance, in Towers of Hanoi and Blocks World, the network depth grows substantially because many apparently valid symbolic actions require additional intermediate motions or object relocations before they become geometrically feasible. In sort clutter, the presence of red obstructing blocks similarly increases the number of graph expansions beyond the minimum number of goal-object transfers.

A second limitation concerns the interpretation of motion planning attempts. In the reported experiments, motion planning failures are treated as evidence that the currently selected transition is not feasible under the available geometric constraints. This assumption is reasonable in simulation when sufficient planning time is allocated and the environment state is known accurately. In real systems, however, a failed planning attempt may arise from several different causes: insufficient planning time, modeling errors, inaccurate object poses, calibration errors, grasp uncertainty, actuation errors, or unexpected contact dynamics. Consequently, the number of motion planning attempts observed in simulation should be interpreted as an optimistic estimate of the effort required in physical deployment.

The benchmarks presented in this work are evaluated in simulation, and this introduces the main sim-to-real limitation of the study. Real-world deployment would introduce perception uncertainty, execution noise, imperfect grasping, object slippage, delays in sensing and actuation, and possible discrepancies between the geometric model and the physical scene. These effects would likely increase the number of low-level planning attempts and may also trigger additional expansions of the AND/OR graph network before a terminal success node is reached. Moreover, physical execution time is typically much larger than planning time. Therefore, even a small increase in the number of failed grasps, failed placements, or inaccurate base motions may have a significant effect on the total task completion time.

From a deployment perspective, this suggests that TMP-IDAN should be coupled with execution monitoring, belief-state estimation, and failure classification mechanisms. In the present formulation, a failure primarily triggers replanning or graph expansion. In a physical robot, it would be useful to distinguish between failures caused by genuine task infeasibility and failures caused by transient execution errors. For example, a grasp failure caused by object slippage should not necessarily lead to the same recovery behavior as a failure caused by an unreachable pose or by an object permanently blocking the target. A natural extension of the framework would therefore associate different classes of failure with different graph-update operations, enabling more selective and efficient recovery.

Another direction concerns uncertainty-aware TMP. The current implementation assumes that the relevant state of the workspace can be represented sufficiently well by the Knowledge Base and Scene Perception modules. In real-world scenarios, however, object poses, object identities, and grasp affordances may only be partially observable. Extending TMP-IDAN with probabilistic state estimates would allow the graph traversal process to take into account not only the nominal feasibility of transitions, but also their expected reliability. This would make it possible to prefer actions that are slightly longer at the symbolic level but more robust at the motion or execution level, thereby providing a more explicit treatment of the task-motion trade-off.

Finally, the benchmark evaluation suggests that AND/OR graph networks are particularly suitable for TMP domains with recursive or repeated structure. Several of the considered problems require the same abstract operation to be applied repeatedly under changing geometric conditions: moving one disk, relocating one block, clearing one obstruction, transferring one object to its goal region, or temporarily undoing and later restoring a previous arrangement. The iterative deepening mechanism exploits this regularity by repeatedly extending the graph network until the goal condition is achieved. This provides an interpretable bridge between symbolic task decomposition and continuous feasibility checking. At the same time, future work should investigate automatic or semi-automatic synthesis of the AND/OR graph structure from higher-level domain descriptions, reducing the manual modeling effort and improving scalability to less structured environments.

## 7 Conclusion

This paper presents a comprehensive evaluation of our TMP algorithm, TMP-IDAN, against five distinct benchmarks proposed by the TMP community. Our approach successfully solves all the benchmarks by changing the structure of the AND/OR graph to meet the specifications of each benchmark. This paper does not claim that our approach is superior to other TMP approaches; rather, our primary objective is to emphasize the adaptability of our approach in addressing diverse TMP applications. Furthermore, to facilitate reproducibility, our code is made publicly available at https://github.com/HosseinKarami1991/TMP_Benchmark.

## Data Availability

The original contributions presented in the study are included in the article/supplementary material; further inquiries can be directed to the corresponding author.

## Footnotes

1 In general, a motion planner fails if no plan exist or because the allotted planning time was insufficient. In this work, we assume that sufficient time is allotted so that a motion planning failure implies the presence of objects obstructing the path to the target object. Thus, if the target is not graspable, then at least one occluding object need to be rearranged to search for a new target-graspable motion plan.
